# Effect of Ramie on the Production Performance of Laying Hens, and the Quality, Nutrient Composition, Antioxidation of the Eggs

**DOI:** 10.3389/fphys.2022.854760

**Published:** 2022-05-30

**Authors:** Xin Wang, Si-Min Peng, Yang Liu, Shuang Liao, Hao-Han Zhao, Guang-Ying Duan, Yong-Mei Wu, Chun-Jie Liu, Yan-Zhou Wang, Tou-Ming Liu, Ying-Hui Li, Zhi-Yong Fan, Si-Yuan Zhu, Hua-Jiao Qiu, Qian Lin

**Affiliations:** ^1^ Institute of Bast Fiber Crops, Chinese Academy of Agricultural Sciences, Changsha, China; ^2^ Hunan Deren Husbandry Technology Co., Ltd., Changde, China; ^3^ College of Animal Science and Technology, Hunan Agriculture University, Changsha, China

**Keywords:** ramie, production performance, egg quality, yolk antioxidation, egg nutrients composition

## Abstract

Ramie (*Boehmeria nivea*), which is rich in protein, fatty acid, vitamins and minerals, has become a potential alternative feed resource for poultry, and has attracted more and more attentions in nutrition research. The objective of this study is to evaluate the effect of dietary ramie at different concentrations on the production performance of the hens, and the quality, nutrient composition, and antioxidation of the eggs. A total of 432 34-week-old Lohmann commercial laying hens were divided into four groups, that were fed with corn-soybean meal-based control diet, control mixed with ramie at concentrations of 3, 6, or 9% separately for 8 weeks. Results showed that dietary ramie did not affect production performance. And egg yolk color gradually deepened as the inclusion levels of ramie increased. Ramie at tested concentration could significantly reduce the content of malondialdehyde (MDA) (*p* = 0.002) and 3% ramie supplementation significantly increased total antioxidative capacity (T-AOC) concentrations in egg yolk compared to the control group (*p* = 0.033). In addition, dietary supplementation with 6% ramie significantly reduced total cholesterol (T-CHO) content (*p <* 0.05) compared with controls. For egg nutrient composition, compared with the control group, the addition of 6% ramie significantly increased (*p <* 0.05) total omega-3 polyunsaturated fatty acids (n-3PUFA) and phenylalanine (Phe) in yolk. In conclusion, dietary inclusion of 6% ramie was most effective in improving the color, antioxidative capability, and reducing T-CHO contents of the egg yolks without any negative impacts on the production performance of the hens.

## Introduction

With the development of economy and living standards, there is an increasing consumption for livestock and poultry products (meat, eggs, milk, *etc*.), which promotes the vigorous development of animal husbandry and demand for feedstuffs. In the past a few years, the lack of feedstuffs has become an important factor restricting the development of the livestock industry due to the shortage of traditional feed resources and the rising prices (Li et al., 2019). To reduce the dependency on the imported feeds and reduce the costs of production, seeking new feed sources with high quality and yield has become a hot spot in the field of animal nutrition (Reda et al., 2022).

Ramie (*Boehmeria nivea*) is a perennial herbaceous plant that belongs to the Urticaceae (Boehmeria) family; it is originated in China, commonly known as “Chinese grass” (Ni et al., 2018). As a traditional textile raw material, only 5% of ramie are utilized, resulting in a great waste of resources (Liu et al., 2013). In recent years, the development and utilization of high-quality unconventional feed resources have attracted much attention. Ramie is a source rich in nutrients, such as proteins, fatty acids (especially oleic acid, palmitic acids and linolerc acid), minerals, and vitamins. It is also well balanced in amino acids with the total amount up to 18.36% (Wang et al., 2019; Wang et al., 2021a). Ramie contains many biologically active compounds in its roots and leaves, such as flavonoids (Rutin, Rhoifolin, beta-ionone) and polyphenols compounds (chlorogenic acid, ferulic acid, caffeic acid), which have been shown to possess antibacterial and anti-inflammation activities *in vitro* (Xiao et al., 2014; Wang et al., 2019).

Previous studies have proved that other unconventional feedstuffs, such as almond hulls, alfalfa and nettle plants, are able to improve egg quality and reduced feed costs (Wang et al., 2021b; Grela et al., 2020; Zhang et al., 2020). It also have been proved that supplementary nettle (*Urtica dioica*), which belong to the same family as ramie, can significantly reduce the lipid metabolism and reduce the total cholesterol, triglyceride contents in blood plasma of mice (Avci et al., 2006). To date, most studies regarding to nettle as an alternative ingredient in laying hens’ feeds have confirmed that it can increase yolk color, decrease total cholesterol contents, improve egg yolk fatty acid composition (Loetscher et al., 2013a; Zhang et al., 2020). Ramie is a plant of *Urticaceae*, and its high nutritional content makes it a potential new material feed. Therefore, the aim of this study was to assess the potential of ramie as a dietary ingredient for the laying hens, by determining the effects of dietary ramie supplementation on the production performance of hens, and the quality, nutrients composition, and oxidative status of the eggs.

## Materials and Methods

All experiment procedures in the present study were approved by the Animal Care Committee of the Institute of Bast Fiber Crops, Chinese Academic of Agricultural Sciences.

### Forage Preparation

The leaves and tender tops of fresh ramie were purchased from Institute of Bast Fiber Crops, Chinese Academy of Agricultural Sciences (Changsha, Hunan, China). The ramie (*Boehmeria nivea cv*. Qingsizhu No.1) was cut at about 60 cm height and immediately dried at 60°C for 4 days by placing in a heat drier room. Then, the dried stems and leaves were crushed to ramie powder using a grinder equipped with 1.5 mm sieve, and were kept in a well-closed and light-resistant place. The nutritional level of ramie powder was crude protein 16.84%, crude fiber 18.7%, calcium 3.3%, total phosphorus 0.30%, lysine 0.64% and methionine 0.04%.

### Experimental Procedure

The experiment was performed at animal experiments base of Institute of Bast Fiber Crops, Chinese Academy of Agricultural Sciences. A total of 432 34-week-old Lohmann Commercial laying hens were randomly divided into four groups, with six replicates of 18 birds each. The hens were raised in ladder cages with two birds each cage. Nine cages with the same diet trough were arranged sequentially as a replicate. After 2 weeks habituation, formal trial started and lasted for 6 weeks. The egg production, body weight, and feed intake of laying hens were measured during the adaptation period to confirm no statistical difference in the production performance among treatments. Laying hens were provided a control diet or 3, 6, 9% ramie powder replacement diet. All diets were formulated to meet recommendations of the National Research Council (NRC) for laying hens (1994) as shown in Table 1. All the diets contain the same ME, crude protein and amino acid *etc*. Free water and feed for all hens. The average temperature was 25 ± 2°C in the laying hens’ house during the experimental period. The light time was according to the standard light procedure of commercial laying hens, 16 h of light per day until the end of the experiment.

**TABLE 1:** Diet formulation and calculated nutrients (air-dried basis, %).

Items	Control	Ramie power supplementation concentration in diets		
		3%	6%	9%
Ingredients, %				
Corn	51.61	51.34	51.23	51.03
Soybean meal	30.78	29.76	28.66	27.59
Rice husk	3.31	2.21	1.09	0.00
Ramie powder	0.00	3.00	6.00	9.00
Oil	2.85	2.50	2.10	1.73
Limestone	8.45	8.19	7.92	7.65
Premix	3.00	3.00	3.00	3.00
Total	100	100	100	100
Nutrient levels (%)				
ME(Mcal/kg)	2.75	2.75	2.75	2.75
Crude protein (%)	16.98	17.00	17.00	17.01
Crude fiber	4.44	4.44	4.44	4.45
Calcium (%)	3.50	3.50	3.50	3.50
Total phosphorus (%)	0.53	0.53	0.53	0.53
Available phosphorus (%)	0.32	0.32	0.32	0.32
Lysine (%)	0.95	0.95	0.94	0.93
Methionine (%)	0.36	0.35	0.35	0.34

a The premix provided the following (per kilogram of complete diet): vitamin A 6000 IU, vitamin D3 2,500 IU, vitamin E 25 mg, vitamin K3 2.25 mg, vitamin B1 1.8 mg, vitamin B2 7 mg, vitamin B6 4 mg, vitamin B12 0.2 mg, D-pantothenic acid 12 mg, nicotinic acid 35 mg, biotin 0.14 mg, folic acid 0.8 mg, Cu (as copper sulphate) 11 mg, Zn (as zinc sulphate) 70 mg, Fe (as ferrous sulphate) 60 mg, Mn (as manganese sulphate) 115 mg, Se (as sodium selenite) 0.30 mg and I (as potassium iodide) 0.4 mg.

^b^Nutrient levels are calculated values.

### Production Performance and Egg Quality

During the test, eggs were picked up at 16:30 pm daily. The feed intake was recorded weekly. laying conditions (egg production and egg weight) of each repetition were recorded in detail. Finally, the egg production, feed conversion ratio, egg weight, daily feed intake and daily egg weight were statistically calculated. Feed conversion ratio was calculated as grams of total feed intake per hen divided by grams of total egg mass per hen, and egg mass was calculated as egg weight multiplied by percentage of egg production. Weights of the albumen, yolk, and shell (12 eggs per replicate) were recorded separately on the last day of the experiment.

Egg quality were determined by parameters of shell strength, shell thickness, Haugh units, egg shaped index, egg yolk index and egg yolk color. Eggshell strength was evaluated using an eggshell force gauge (model II, Robotmation Co., Ltd., Tokyo, Japan). Shell thickness was measured using a vernier caliper and was taken as the mean value at three points: the two narrow ends and the middle part of the egg. Haugh unit and Yolk color were determined using an egg multi-tester (EMT-7300, Robotmation, Tokyo, Japan). Egg-shaped index meter (FHK, Mingao Instrument Equipment, Nanjing, China) was used to evaluate the egg shape index. In order to keep the egg yolk intact, the egg yolk diameter and height were accurately measured by vernier caliper. Each egg was measured three times, and the average value was taken. The ratio of egg yolk height to egg yolk diameter was egg yolk index.

### Yolk Antioxidation

Twenty-four eggs (one eggs/replicate) were randomly selected to analyze the antioxidant index of the yolk on the last day. Egg yolk was separated by manual separator, and then placed at −20°C for testing. Total antioxidative capacity (T-AOC) was determined by detecting the sample’s ability to reduce Fe^3+^ to Fe^2+^ at 520 nm (Sun et al., 2015). Glutathione peroxidase (GSH-Px) activity were determined by measuring the oxidation rate of quantitative glutathione to oxidized glutathione at 412 nm (Fouad et al., 2016). Total superoxide dismutase (T-SOD) activity was determined by hydroxylamine method (Liu H. N. et al., 2014), catalase (CAT) activity was determined by ammonium molybdate method (Sun et al., 2015), and malondialdehyde (MDA) level was determined by thiobarbituric acid method (Yang et al., 2010). The above indicators were determined with the kits purchased from Nanjing Jiancheng Bioengineering Institute in strict accordance with the instructions.

### Egg Nutrient Composition

During the sixth week of the study, 432 eggs were collected from each treatment (18 eggs/replicate) to analyze the nutrient composition of the egg. Among those, 144 eggs (36 eggs/treatment) were chosen for whole eggs chemical analysis, including moisture, crude protein (CP) (N × 6.25), ether extract (EE), and ash content (ASH), using methods developed by the AOAC (Association of Official Analytical Chemists). 144 eggs (36 eggs/treatment) were selected for the analysis of the amino acid content of whole eggs. The contents of 17 free amino acid (g/100 g) were determined by S433D amino acid automatic analyzer (Sykam, Germany) followed the method described by the regulations and requirements of determination of Amino Acids in foods (China, GB 5009.124-2016, China Food and Drug Administration, 2016). The content of Tryptophan in Freeze - dried Powder was individually determined by reversed-phase liquid chromatography (RP - HPLC). The remaining 144 eggs (36 eggs per treatment) were characterized for the biochemical parameters analysis in egg yolk. The total cholesterol content (mmol/gyolk) in egg yolk was measured by cholesterol oxidase method. The determination was carried out with the kit of Nanjing Jiancheng Institute of Biological Engineering in strict accordance with the instructions. The fatty acid composition of the yolk was analyzed using an Agilent 7890A gas chromatography (Agilent Technologies, Wilmington, United States) according to the regulations and requirements of Determination of National Food Safety Standard: Determination of Fatty Acids in Food (China, GB 5009.168-2016, China Food and Drug Administration, 2016).

### Statistical Analysis

The data were tested by ANOVA for the control group and experiment groups with Statistical Packages for Social Science 18.0 (SPSS 18.0) software. Orthogonal polynomial contrasts were used to determine linear and quadratic responses of Lohmann commercial laying hens to different level ramie powder. For significant effects, means were compared by Duncan’s multiple comparison test to determine specific differences between means. Statistical significance was assigned at *p* < 0.05. The *p* values between 0.05 and 0.10 were considered as having trends in difference.

## Results

### Production Performance

The effect of dietary supplementation of ramie on the production performance of laying hens is presented in Table 2. No mortality was found during the 6 weeks experimental period. The dietary ramie supplementation had no effects on feed conversion ratio (FCR), egg weight, feed intake or the egg mass. There was an increasing trend in the average egg production in the ramie treated groups compared to the control throughout the whole experimental period (quadratic, 0.05 < *p* < 0.10).

**TABLE 2:** Effects of dietary ramie supplementation on laying hen production performance.

Items	Control	Ramie supplementation concentration in diets			SEM	*p*-value		
		3%	6%	9%		ANOVA	Linear	Quadratic
Egg laying rate (%)	89.05	94.16	94.82	89.34	1.053	0.090	0.651	0.015
FCR	2.20	2.12	2.13	2.21	0.038	0.852	0.990	0.386
Egg weight (g)	56.51	56.59	56.91	56.91	0.139	0.640	0.225	0.913
Daily feed intake (g)	114.20	109.86	111.77	113.91	1.596	0.761	0.918	0.315
Egg mass per day (g)	51.97	51.94	52.41	51.48	0.585	0.979	0.828	0.803

^a,b^Means in the same row with different superscript letters indicate differences (*p* < 0.05); The *p* values between 0.05 and 0.10 were considered as having trends in difference.

FCR, food conversion ratio; SEM, standard error of the mean.

### Egg Quality

**FIGURE 1:**
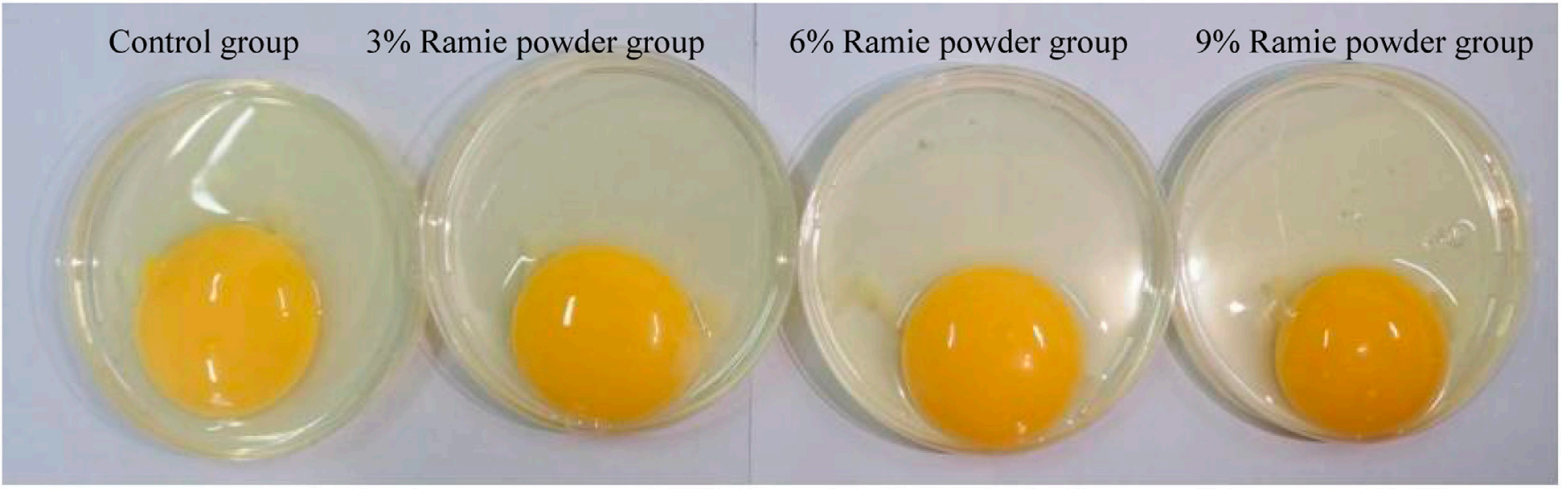
Effect of diet on egg yolk color of laying hens (40 weeks of age). From left to right: control, 3, 6, 9% ramie supplementation diets in turn.

As shown in Table 3, with the increase of dietary ramie content, egg yolk color gradually deepened, and ramie supplementation at 6%, 9% significantly increased the egg yolk color compared to the control (linear, *p* < 0.001; Figure 1). Ramie powder supplement of 9% increased the egg yolk index (linear, *p* = 0.034). None of the other egg quality traits (egg weight, egg strength, shell thickness, Haugh unit, shell weight, yolk weight, and protein weight) were affected by the dietary ramie supplementation (*p* > 0.10).

**TABLE 3:** Effects of dietary ramie supplementation on the egg quality of laying hens.

Items	Control	Ramie supplementation concentration in diets			SEM	*p*-value		
		3%	6%	9%		ANOVA	Linear	Quadratic
Egg weight (g per egg)	58.04	59.39	59.69	59.62	0.548	0.797	0.422	0.582
Eggshell strength (kgf)	4.58	4.62	4.61	4.56	0.049	0.976	0.936	0.665
Eggshell thickness (mm)	0.38	0.40	0.39	0.39	0.002	0.467	0.964	0.154
Haugh unit	75.83	76.18	76.84	77.57	0.587	0.767	0.303	0.882
Egg shape index	1.34	1.33	1.34	1.33	0.005	0.907	0.561	1.000
Yolk Index	0.36^b^	0.36^b^	0.37^ab^	0.39^a^	0.005	0.034	0.009	0.172
Yolk color	4.20^b^	4.47^b^	5.30^a^	5.63^a^	0.152	<0.001	<0.001	0.883
shell weight (g per egg)	5.75	5.68	5.72	5.80	0.084	0.970	0.810	0.683
yolk weight (g per egg)	15.84	16.17	16.54	16.33	0.196	0.681	0.332	0.522
Protein weight (g per egg)	36.71	36.97	38.41	37.49	0.463	0.638	0.493	0.438

^a,b^Means in the same row with different superscript letters indicate differences (*p* < 0.05).

SEM, standard error of the mean.

### Yolk Antioxidation

Eggs from laying hens fed 3% ramie diet had significantly higher T-AOC in the yolk (*p* < 0.05) compared to the ones from the control group (Table 4). The antioxidative indices of the egg yolks was shown in Table 4. Ramie supplementation at 3% significantly increased the T-AOC level compared to the ones in the control groups (*p* = 0.033), and significant quadratic relation was nocited between them (*p =* 0.024). Moreover, the dietary supplementation with ramie significantly reduced the content of MDA in egg yolk (*p* = 0.002), and significant linear and quadratic relationships were found between the ramie concentration and MDA content (*p* = 0.016 and 0.004 respectively). However, not effect was noticed between the level of ramie on the contents of T-SOD, GSH-Px, or CAT (*p* > 0.05).

**TABLE 4:** Effects of dietary ramie supplementation on Yolk Antioxidation Indices.

Items	Control	Ramie supplementation concentration in diets			SEM	*p*-value		
		3%	6%	9%		ANOVA	Linear	Quadratic
T-AOC (U/ml)	12.11^b^	15.98^a^	13.85^ab^	13.56^ab^	0.490	0.033	0.565	0.024
T-SOD (U/g)	229.76	273.19	248.68	266.94	14.443	0.737	0.524	0.680
GSH-Px (U/g)	306.05	333.74	342.08	360.77	15.134	0.654	0.223	0.877
CAT (U/g)	30.43	31.86	36.76	35.95	1.273	0.227	0.065	0.655
MDA (nmol/g)	77.68^a^	62.20^b^	66.05^b^	66.74^b^	1.660	0.002	0.016	0.004

^a,b^Means in the same row with different superscript letters indicate differences (*p* < 0.05).

T-AOC, total antioxidative capacity; T-SOD, total Superoxide dismutase; GSH-Px, glutathione peroxidase; CAT, catalase; MDA, malondialdehyde; SEM, standard error of the mean.

### Nutritional Composition of the Eggs and Total Cholesterol Content of the Egg Yolk

The contents of total cholesterol in egg yolk was significantly decreased by 12.27%, 15.80% in 3 and 6% ramie treated groups compared to the control groups (*p* = 0.043), with significant linear and quadratic relationships (*p* = 0.040 and 0.013 respectively). But there were no differences in the content of moisture, CP, EE or Ash of eggs among the groups (Table 5).

**TABLE 5:** Effects of dietary ramie supplementation on Nutritional Composition of Whole Egg and Total Cholesterol Content in Egg Yolk.

Items	Control	Ramie supplementation concentration in diets			SEM	*p*-value		
		3%	6%	9%		ANOVA	Linear	Quadratic
Moisture (%)	75.73	75.10	75.54	75.12	0.201	0.635	0.468	0.740
CP (%)	12.22	12.89	12.49	12.35	0.128	0.181	0.685	0.070
EE (%)	7.19	8.38	8.07	7.70	0.189	0.159	0.517	0.052
Ash (%)	0.78	0.73	0.74	0.77	0.009	0.290	0.965	0.091
T-CHO (mmol/gyolk)	5.95^a^	5.22^b^	5.01^b^	5.36^ab^	0.110	0.043	0.040	0.013

^a,b^Means within a row with no common superscripts differ significantly (*p* < 0.05).

CP, crude protein; EE, ether extract; Ash, ash content; T-CHO, total cholesterol; SEM, standard error of the mean.

### Fatty Acids Composition of Egg Yolk

The fatty acids composition of the egg yolks was shown in Table 6. Compared to the control groups, 6% ramie supplement diets significantly increased the content of eicosadienoic acid (*p* = 0.040), ALA (*p* = 0.033), LA (*p =* 0.004), PUFA (*p* = 0.002), n-3 PUFA (*p* = 0.003), and EFA (*p* = 0.004) in egg yolks. Additionally, 6 and 9% ramie supplementation significantly increased the content of LA (*p =* 0.004), PUFA (*p* = 0.002), and EFA (*p* = 0.004) in the egg yolks compared to the ones in the control groups. Moreover, significant linear relationships were found between the levels of ramie supplementation and eicosadienoic acid (*p* = 0.046), LA (*p* = 0.004), PUFA (*p* = 0.002), and EFA (*p* = 0.004); and quadratic relationships were found between the levels of ramie supplementation and ALA (*p* = 0.033), LA (*p* = 0.002), PUFA (*p* = 0.001), n = 3 PUFA (*p* = 0.001), and EFA (*p* = 0.002).

**TABLE 6:** Effects of dietary ramie supplementation on fatty acid composition in egg yolk (g/100 g FA).

Items	Control	Ramie supplementation concentration in diets			SEM	*p*-value		
		3%	6%	9%		ANOVA	Linear	Quadratic
C12:0	0.00	0.00	0.01	0.01	0.004	0.452	0.157	0.974
C14:0	0.33	0.34	0.33	0.35	0.009	0.867	0.652	0.733
C15:0	0.05	0.05	0.04	0.05	0.003	0.242	0.482	0.595
C16:0	25.38	24.77	24.40	25.09	0.331	0.781	0.612	0.404
C17:0	0.13	0.15	0.15	0.15	0.005	0.562	0.302	0.365
C18:0	8.68	8.29	9.02	8.79	0.171	0.609	0.579	0.782
C20:0	0.03	0.00	0.01	0.00	0.004	0.079	0.076	0.285
C22:0	0.05	0.03	0.03	0.02	0.007	0.291	0.075	0.919
C14:1	0.09	0.08	0.07	0.07	0.007	0.884	0.500	0.712
C16:1	3.54	3.26	2.72	3.12	0.287	0.812	0.484	0.616
C20:1	0.23	0.20	0.21	0.21	0.005	0.395	0.210	0.371
C18:1n-9t	0.14	0.12	0.14	0.13	0.004	0.414	0.640	0.976
C18:1n-9c (oleinic acid)	39.90	38.70	37.51	38.65	0.447	0.418	0.332	0.224
C20:2 (eicosadienoic acid)	0.16^b^	0.17^b^	0.22^a^	0.18^ab^	0.008	0.040	0.046	0.081
C22:6n-3 (DHA)	1.18	1.28	1.40	1.19	0.051	0.435	0.636	0.172
C18:3n-3 (ALA)	0.58^b^	0.66^b^	0.87^a^	0.64^b^	0.041	0.033	0.108	0.033
C18:3n-6 (GLA)	0.13	0.15	0.13	0.17	0.008	0.350	0.216	0.506
C18:2n-6c (LA)	13.57^c^	19.24^b^	22.17^a^	18.44^b^	1.196	0.004	0.004	0.002
C20:3n-6 (eicosatrienoic acid)	0.30	0.25	0.26	0.28	0.013	0.532	0.577	0.210
C20:4n-6 (AA)	2.75	3.02	2.80	3.00	0.060	0.322	0.371	0.657
SFA	34.64	33.63	33.99	34.45	0.278	0.612	0.754	0.238
MUFA	43.37	42.83	41.75	43.16	0.720	0.919	0.842	0.619
PUFA	18.75^c^	24.78^b^	28.10^a^	24.03^b^	1.286	0.002	0.002	0.001
UFA	64.55	66.43	66.61	66.44	0.382	0.148	0.076	0.135
n-6 PUFA	20.65	21.41	21.64	20.52	0.745	0.965	0.986	0.648
n-3 PUFA	1.58^b^	1.94^b^	2.57^a^	1.82^b^	0.117	0.003	0.078	0.002
n-6/n-3 PUFA	11.69	10.73	9.87	11.35	0.362	0.354	0.558	0.140
EFA	14.21^c^	20.04^b^	23.24^a^	19.25^b^	1.253	0.004	0.004	0.002

^a–c^Means in the same row with different superscript letters indicate differences (*p* < 0.05).

SEM, standard error of the mean; LA, linoleic acid; AA, arachidonic acid; ALA, α-linolenic acid; DHA, docosahexaenoic acid; SFA, saturated fatty acid; MUFA, monounsaturated fatty acid; PUFA, polyunsaturated fatty acid; EFA, essential fatty acid.

1 SFA level were calculated as C15:0 + C16:0 + C17:0 + C18:0.

2 MUFA levels were calculated as C16:1 + C17:1 + C18:1n-9 + C20:1n-9 + C22:1n-9 + C24:1n-9.

3 PUFA levels were calculated as C18:2n-6 + C18:3n-6 + C18:3n-3 + C20:2 + C20:3n-6 + C20:3n-3 + C20:4n-6 + C20:5n-3 + C22:6n-3.

4 n-6 was calculated as C18:2n-6 + C18:3n-6 + C20:3n-6 + C20:4n-6.

5 n-3 was calculated as C18:3n-3 + C20:3n-3 + C20:5n-3 + C22:6n-3.

6 EFA was calculated as ALA + LA.

### Amino Acid Content of the Eggs

Eggs from hens fed ramie-supplemented diet exhibited a higher EAA/NEAA and EAA/TAA value compared to the ones in the control groups (*p* = 0.037 and 0.031, respectively). There was a tendency of increasing the content of EAA in whole eggs by ramie supplementation (*p* = 0.055). Additionally, as compared to the ones in the controls, 6% ramie supplementation significantly increased phenylalanine content (*p* = 0.026). Significant linear relationships were notice between the level of ramie supplementation and the contents of phenylalanine and EAA/TAA (*p* = 0.019 and 0.038, respectively).

## Discussion

Ramie leaves, which are commonly used for medicinal and edible purposes, are effective in reducing serum cholesterol and improving meat quality of farmed animals (Avci et al., 2006; Tang et al., 2021). However, there is only limited research on the effects of dietary ramie supplementation on laying hens. Thus, the aim of this study is to investigate whether the production performance, egg quality traits, yolk antioxidation indices and egg nutrients composition of laying hens are affected by the dietary supplementation of ramie. Ramie had no adverse effect on the overall production performance of hens in the present study. Many researches showed that the egg production increased in response to the nettle supplementation, such as *Urtica dioica*, *Urtica cannabina* and *Urtica fissa* (Loetscher et al., 2013a; Zhang et al., 2020; Liu et al., 2010). Ramie as a perennial dicotyledon of the Urticaceae family (Wei et al., 2014), might possess the same effect as nettle. Previous studies reported that egg production remained unchanged (Wang and Jie, 2012) or even tended to increase (Luo et al., 1989) with the supplement of ramie. In line with these studies, the supplementation of 3 and 6% ramie powder tended to increase the egg production in our study. Ramie is rich in cellulose, flavonoids compounds, polyphenol compounds, vitamin C, and minerals. It might partially because of the antioxidant properties of ramie contribute to the improved production performance. Recent studies showed that adding flavonoids to the diet can improve the performance of laying hens by improving the body’s antioxidant capacity, reducing the occurrence of oxidative stress and promoting the absorption of nutrients in the intestine of laying hens (Brisibe et al., 2008; Galal et al., 2008; Seven, 2008; Liu L. L. et al., 2014; Zhu et al., 2021).

Yolk color is an important quality trait of eggs which is extremely critical to consumers. In our study, the yolk color was enhanced significantly by adding a certain amount (6–9%) of ramie. Similar results were also observed that yolk color gradually deepened as the addition levels of *urtica dioica* or *urtica cannabina* in diets (Like *Boehmeria nivea*, they are Urtica plants) (Loetscher et al., 2013a; zhang et al., 2020). *U. dioica* was found to be equally as effective as synthetic pigmentation. It was shown that the inclusion of *U. dioica* in food increases the enrichment and bioaccessibility of lutein and *β*-carotene (provitamin A carotenoids) at the duodenum digestion stage (Kregiel et al., 2018). Lutein was considered an important natural pigment for ameliorating egg yolk and broiler skin color, which could be deposited in egg yolks (Hencken, 1992; Loetscher et al., 2013a; Loetscher et al., 2013b). Besides, nettle was reported to be rich in yellow-colored xanthophylls, with lutein (184 μg/g) being the predominant compound, followed by β-carotene (6.7 μg/g) (Marchetti et al., 2018). And xanthophylls were found to be absorbed in the digestive tract and deposited in subcutaneous fat and yolk leading to higher yolk color scores (Wen et al., 2019). Therefore, the higher yolk color observed in ramie (6–9%) treated groups in the present study might be due to the high levels of effective polar xanthophylls, such as lutein. Additionally, supplemental antioxidants to layer feed could also improve the yolk color (Yuan et al., 2016). Neohesperidin dihydrochalcone is a hydrogenated flavonoid derivative and has antioxidant, antimicrobial and anti-inflammatory properties in animals (Yeomans et al., 2007; Marti et al., 2008). It could improve the egg yolk color because it reduced the effects of lipid peroxides and free radicals on organisms (Zhu et al., 2021). It could also be possible that ramie (rich in flavonoid constituent) prevented the lutein from being oxidized and therefore increased the pigment deposition (Wang et al., 2019).

Egg yolk index can be used to measure the egg yolk nutrient concentration and egg freshness, and the higher egg yolk index means better egg processing grade and edible value. Previous studies showed that adding 2% nettle powder to the diet of laying hens could significantly increase the yolk index (Mansoub, 2011). Our study showed that egg yolk index was significantly increased in the 9% ramie group. The increase in egg yolk index might be related to the stability of yellow pigment in the lipid molecules located in egg yolk membrane, as ramie prevent the occurrence of the oxidative stress.

Nettle could also reduce lipid peroxidation (serum MDA content decreased) and liver enzyme activity in CCl4-treated rats, and improved the activity of antioxidant defense system (Kanter et al., 2003). Our study showed that ramie supplmentation significantly reduced MDA content in egg yolk. In addition, supplementation of 3% ramie powder significantly increased the T-AOC capacity of egg yolk. These indicated that by adding ramie powder, the antioxidant capacity of egg yolk was improved.

Total cholesterol content in eggs can be reduced by adding natural plants, cellulose and trace elements (Zhang et al., 2010). The addition of 15% *Urtica cannabina* in the diet of laying hens could significantly reduce the cholesterol content in yolk, without affecting the CP and EE content in eggs (Zhang et al., 2020). The addition of 6% *Urtica dioica* could significantly reduce the cholesterol content in quail egg yolk (Moula et al., 2019). Our study showed that ramie supplementation had no significant effect on the nutritional composition of whole eggs, but the total cholesterol concentration in yolk with 3 and 6% ramie treated groups was significantly decreased. This might be due to the phytosterol components in ramie which could reduce the absorption of cholesterol in intestinal tract, thereby lowering cholesterol levels in the blood, and subsequently, in animal products (Avci et al., 2006).

N-3PUFA, including ALA, EPA and DHA, has been recognized for its beneficial effects on the growth, health and immune function of humans and animals (Lee et al., 2009; Chen et al., 2014; Zhang et al., 2020). Numerous fatty acid desaturases played key roles in synthesizing PUFA. Several desaturases are absent in animals and humans, such as delta-12 and delta-15 desaturases (Lee et al., 2016). Thus, ALA must be obtained from the diet and be converted into EPA and DHA by delta-6 desaturase catalyzed dehydrogenation and the addition of two carbons by an elongase (Fraeye et al., 2012). Dietary supplementation with fresh nettle reduced the ratio of n-6/n-3 and increased the total n-3PUFA in yolk (Zhang et al., 2020). Similarly, it was reported that dietary supplementation with fresh nettle increased the contents of linoleic acid and linolenic acid, improved the proportion of PUFA and n-3PUFA and reduced the ratio of n-6/n-3 in the breast meat of broiler (Stojcic et al., 2016). Moreover, it was showed that the fat metabolism of pigs was modulated by 500 mg/kg nettle extract in diet, that the MUFA was decreased and PUFA was increased in muscle fat (Szewczyk et al., 2006). The composition of fatty acids stored in monogastric animals indicated the possibility that the lipid and fatty acid composition of poultry eggs could be altered by the diet (Kouassi et al., 2020). In the present study, 6% ramie significantly increased the content of icosaic acid and ALA in egg yolk, and significantly increased the content of n-3PUFA. In addition, the ramie supplementation was associated with a significant elevation in the LA, PUFA and EFA proportion compared to the control diet. This might be related to the rich sources of essential fatty acids in ramie leaves. ALA is the main fatty acid accounting for 40.7% of the fatty acids in mature leaves (Guil-Guerrero et al., 2003). Hence, eggs might receive the high content of n-3PUFA when the laying hens were fed by diets rich in n-3PUFA (Wen et al., 2019; Grˇcevi´c et al., 2019). Another possible explanation for the higher total n-3PUFA content in the egg yolks of the groups treated with ramie could be because of the protective function of the antioxidative compounds such as lutein, tocopherol, flavonoids and phenolic compounds. Nettle is an abundant source of lutein, which has been considered to be an important natural pigment for improving egg yolk and broiler skin color (Loetscher et al., 2013b; Zhang et al., 2020). Lutein and flavonoids compounds are potent antioxidants due to their free radical quenching activities (Irgin et al., 2016; Kamoshita et al., 2016). In previous study, the addition of marigold powder (rich in lutein) to the laying hens’ feed significantly increased egg lutein content, which helped to preserved a higher content of DHA in the yolks. Adding *Urtica cannabina* to the feed of laying hens (or due to the presence of antioxidant compounds) can maintain high DHA content in egg yolk (Zhang et al., 2020). Based on these results, we assume that the richness of antioxidants in ramie had an impact on the conservation of PUFA in the lipids of the egg yolks by protecting them from oxidation and damage, thereby maintaining a higher level of PUFA in the ramie group. Collectively, these findings indicated that, as an important n-3 PUFA source, ramie contributed to the excessive production of n-3 PUFA in the eggs.

As one of the main animal products, eggs are welcome in the market as excellent protein source (Perić et al., 2011). The nutritional value of protein mainly depended on the variety and content of the amino acids. Sufficient content of essential amino acid and balance amino acid composition played an extremely important role in the nutritional value and edible flavor of food (Wu et al., 2013). Many studies showed that the nutrition and flavor of eggs were affected by factors such as dietary nutrients and feeding methods (Perić et al., 2011; Bashir et al., 2015; Dong et al., 2018; Bagheri et al., 2019). The tender stems and leaves of ramie contained high level of crude protein. After Boer goats feeding with silage of ramie tender stems and leaves, the content of umami amino acids and proline in the mutton were increased, which was beneficial in the umami taste, the protein bioavailability, and the quality of mutton (Gao et al., 2016). The addition of silage ramie as a source of roughage to Simmental beef cattle diets not only maintained the quality and nutritional value, but also improved the composition of flavor amino acids (umami amino acids, bitter amino acids, and sour amino acids) in beef (Yang et al., 2017). The results of the present experiment show that supplementation of ramie had no effect on the content of umami amino acids in eggs. It was different from previous researches, and the possible explanations might be the different processing methods of ramie before feeding (without silage), and the difference in experiment animals, who possessed different digestion and absorption capabilities. It was reported that 9% canola meal supplementation in diet increased the total amino acid content in eggs of Roman laying hens (Wang et al., 2013). As in the present study, supplementation of 6% ramie significantly increased phenylalanine content in the whole eggs, and different levels of ramie significantly increased EAA/TAA and EAA/NEAA in whole eggs. The trend of increased content of essential amino acids in the whole eggs in ramie treated groups might be because the amino acid content in ramie leaves is quite rich (EAA/TAA value was greater than 40%, and EAA/NEAA value was greater than 69%) (Sun et al., 2013). After the essential amino acids in ramie leaves being digested and absorbed by laying hens, they were deposited in eggs. It was also possible that ramie contains a variety of active substances, which improves intestinal health and nutrient absorption, thereby increased the content of essential amino acids in eggs (Graf, 1992; Nardini et al., 1995; Van Acker et al., 2000; Miyake et al., 2003; AOAC International, 2005).

## Conclusion

In summary, with a dietary supplement of ramie, no negative effects on egg production and egg quality in laying hens were found, but increase of the antioxidant capacity of the egg yolk and the nutritional content of the whole egg were achieved. The yellow color of egg yolk deepened as the increase of ramie content, and 9% ramie supplements contributed the darkest egg yolk. Compared with other groups of laying hens, supplementation of 6% ramie in the diet significantly reduced the total cholesterol content in the egg yolk; and increased the content of n-3 polyunsaturated fatty acids and essential amino acids in the whole eggs. However, how the biologically active ingredients in ramie affect the content of unsaturated fatty acids in eggs needs further research. These findings support the potential application of ramie as a dietary supplementation for laying hens (Table 7).

**TABLE 7:** Effects of dietary ramie supplementation on Amino Acid Content in Whole Egg (g/100 g DW).

Items	Ingredient	Control	Ramie supplementation concentration in diets			SEM	*p*-value		
			3%	6%	9%		ANOVA	Linear	Quadratic
nonessential amino acid	Asp	1.04	1.06	1.07	1.05	0.008	0.508	0.541	0.189
	Tyr	0.38	0.40	0.41	0.40	0.006	0.647	0.402	0.347
	Ser	0.52	0.54	0.55	0.52	0.006	0.179	0.652	0.040
	Glu	0.94	0.94	0.98	0.95	0.008	0.262	0.299	0.425
	Gly	0.42	0.41	0.43	0.42	0.005	0.629	0.758	0.930
	Ala	0.65	0.65	0.67	0.65	0.007	0.463	0.535	0.574
	Cys	0.44	0.47	0.46	0.45	0.006	0.397	0.513	0.193
	Arg	1.25	1.21	1.14	1.17	0.026	0.404	0.151	0.524
	Pro	0.53	0.56	0.59	0.49	0.018	0.302	0.632	0.094
	His	0.47	0.49	0.47	0.45	0.007	0.263	0.171	0.205
essential amino acid	Met	0.24	0.29	0.27	0.28	0.009	0.406	0.293	0.292
	Val	0.47	0.50	0.49	0.49	0.008	0.409	0.287	0.256
	Lys	0.78	0.82	0.80	0.80	0.009	0.449	0.605	0.249
	Ile	0.40	0.40	0.39	0.40	0.007	0.981	0.993	0.965
	Phe	0.34^b^	0.36^ab^	0.37^a^	0.36^ab^	0.005	0.026	0.019	0.050
	Leu	0.67	0.69	0.70	0.70	0.008	0.648	0.256	0.632
	Trp	0.20	0.23	0.23	0.24	0.009	0.569	0.193	0.722
	Thr	0.43	0.45	0.45	0.43	0.005	0.210	0.394	0.056
	EAA	3.42	3.72	3.68	3.60	0.045	0.055	0.152	0.023
	NEAA	6.64	6.72	6.75	6.55	0.048	0.507	0.719	0.166
	FAA	4.27	4.27	4.29	4.28	0.150	0.993	0.831	0.946
	TAA	10.05	10.44	10.43	10.15	0.082	0.223	0.608	0.047
	EAA/NEAA	0.51^b^	0.56^a^	0.54^a^	0.55^a^	0.006	0.037	0.061	0.061
	EAA/TAA	33.97^b^	35.66^a^	35.26^a^	35.41^a^	0.234	0.031	0.038	0.066
	FAA/TAA	41.56	41.78	41.52	41.75	0.716	0.968	0.864	0.954

^a,b^Means within a row with no common superscripts differ significantly (*p* < 0.05).

Asp, aspartic acid; Tyr, tryptophan; Ser, serine; Glu, glutamic acid; Gly, glycine; Ala, alanine; Cys, cysteine; Arg, arginine; Pro, proline; His, histidine; Met, methionine; Val, valine; Lys, lysine; Ile, l-isoleucine; Phe, phenylalanine; Leu, leucine; Trp, tryptophan; Thr, threonin; EAA, essential amino acid; NEAA, nonessential amino acid; FAA, umami amino acids; TAA, total amino acids.

## Data Availability

The original contributions presented in the study are included in the article/Supplementary Material, further inquiries can be directed to the corresponding authors.
